# NBM-T-L-BMX-OS01, Semisynthesized from Osthole, Is a Novel Inhibitor of Histone Deacetylase and Enhances Learning and Memory in Rats

**DOI:** 10.1155/2013/514908

**Published:** 2013-03-28

**Authors:** Ying-Chen Yang, Chia-Nan Chen, Carol-Imei Wu, Wei-Jan Huang, Tsun-Yung Kuo, Ming-Chung Kuan, Tung-Hu Tsai, Jing-Shi Huang, Chung-Yang Huang

**Affiliations:** ^1^Department of Biotechnology and Animal Science, College of Bioresources, National Ilan University, Ilan 260, Taiwan; ^2^New Drug Research & Development Center, NatureWise Biotech & Medicals Corporation, Taipei 112, Taiwan; ^3^Graduate Institute of Pharmacognosy, Taipei Medical University, Taipei 110, Taiwan; ^4^Institute of Traditional Medicine, School of Medicine, National Yang-Ming University, Taipei 112, Taiwan; ^5^Graduate Institute of Acupuncture Science, China Medical University, Taichung 404, Taiwan; ^6^Department of Education and Research, Taipei City Hospital, Taipei 103, Taiwan

## Abstract

NBM-T-L-BMX-OS01 (BMX) was derived from the semisynthesis of osthole, isolated from *Cnidium monnieri* (L.) Cuss., and was identified to be a potent inhibitor of HDAC8. This study shows that HDAC8 is highly expressed in the pancreas and the brain. The function of HDAC8 in the brain has not been adequately studied. Because BMX enhances neurite outgrowth and cAMP response element-binding protein (CREB) activation, the effect of BMX on neural plasticity such as learning and memory is examined. To examine declarative and nondeclarative memory, a water maze, a passive one-way avoidance task, and a novel object recognition task were performed. Results from the water maze revealed that BMX and suberoylanilide-hydroxamic-acid-(SAHA-) treated rats showed shorter escape latency in finding the hidden platform. The BMX-treated animals spent more time in the target quadrant in the probe trial performance. An analysis of the passive one-way avoidance results showed that the BMX-treated animals stayed longer in the illuminated chamber by 1 day and 7 days after footshock. The novel object recognition task revealed that the BMX-treated animals showed a marked increase in the time spent exploring novel objects. Furthermore, BMX ameliorates scopolamine-(Sco-) induced learning and memory impairment in animals, indicating a novel role of BMX in learning and memory.

## 1. Introduction

Chromatin, a densely packed higher-order complex structure containing DNA and histone proteins, is present in eukaryotic cells. Epigenetic modifications such as acetylation, methylation, and phosphorylation of histones are important in chromatin remodeling and the modulation of gene expression. All of these epigenetic modifications of amino acids may occur on the N-terminal tail of histones. There are five major families of histones: H1/H5, H2A, H2B, H3, and H4. Histones H2A, H2B, H3, and H4 are known as the core histones, while histones H1 and H5 are known as the linker histones [1]. Structural modifications of histones, especially H3 and H4, mainly occur in acetylation or deacetylation of the N-terminal tail through histone acetyltransferases (HATs) and histone deacetylases (HDACs). Mammalian HDACs are divided into 4 classes based on their function and structural homologies to yeast HDACs. Class I HDACs are HDAC1, -2, -3, and -8. Class II HDACs are HDAC4, -5, -6, -7, -9, and -10. Class III HDACs are SIRT 1–7, nicotinamide-adenine-dinucleotide- (NAD^+^-) dependent, and are involved in longevity. Class IV HDACs include HDAC11, which is Zn^2+^-dependent [2]. HDACs modulate both histone and nonhistone proteins. The nonhistone proteins, such as transcription factors (e.g., p53, STAT1, or STAT3), cytoskeleton proteins (e.g., *α*-tubulin), and other cellular proteins (e.g., HSP90 or KU70), are targets for reversible acetylation as well. Acetylation of target nonhistone proteins has been suggested to influence protein stability, activity, localization, and binding efficiency with cofactors [3, 4].

Long-term memory formation relies on structural changes and gene transcription. Enriched environment stimulation is well known to enhance memory formation, and it has now been proven that enriched environment-activated histone acetylation reverses memory impairment in p25-overexpressed brain-atrophic mice [5]. Sodium butyrate as a pandemic HDAC inhibitor mimics the effect of an enriched environment in restoring p25 transgenic mice memory as well [5]. This suggests that HDAC has a role in cognitive function. In brief, regarding the effect of Class I HDACs on memory, inactivation of HDAC1 by p25 precedes Alzheimer's disease and neuronal death [6]. Overexpression of HDAC1 does not affect memory in mice, whereas overexpression of HDAC2 impaired memory formation. Knockout of HDAC2 improves conditioned fear memory and synaptic plasticity [7]. The role of HDAC1 in memory is contrary to that of HDAC2. HDAC3-deficient mice showed longer retention time for novel objects in a novel object recognition experiment, indicating that HDAC3 also plays a negative role in memory formation [8]. Regarding HDAC8, few reports exist on its role in memory formation or neural plasticity. 

To date, HDACis found to improve learning and memory are pan-HDAC inhibitors such as trichostatin A (TSA) [9], sodium butyrate [9], suberoylanilide hydroxamic acid (SAHA) [10], and valproic acid [11]. An increasing number of studies have implicated a role for SAHA in cognitive function and brain-related diseases [7, 12–14]. SAHA has been shown to cross the blood-brain barrier (BBB), increase histone acetylation in the brain, and reverse contextual memory deficits in a mouse model of Alzheimer's disease [15]. SAHA was shown to inhibit the deacetylase activity of Class I HDAC1, -2, -3, and -6 with little inhibition of HDAC8 [16]; SAHA enhances memory predominantly through inhibition of HDAC2 activity [7]. SAHA was approved by the US FDA for treating cutaneous T-cell lymphoma (CTCL) in 2006. Although SAHA shows potential in treating neurodegenerative diseases associated with cognitive impairment, however, knockdown of HDAC2 results in fragmented, detached cells, suggestive of apoptosis [17]. In addition, SAHA also inhibits HDAC1, and loss of HDAC1 leads to neurotoxicity [6, 18, 19]. SAHA might damage neuronal cells through inhibition of HDAC1 or HDAC2 enzyme activity which cannot be ruled out [20]. In this study, SAHA was selected among numerous HDAC inhibitors as a control because it is well studied and is regarded as the potential drug to treat neurodegenerative diseases and has less selectivity on HDAC8. Because of the possibility of cell damage from pan-HDACis, identifying a selective HDACi in learning and memory is a worthy pursuit. 

Amnesia is a major symptom of aging and dementia in elderly people and Alzheimer's disease patients. Traditional Chinese medicines such as Tianma, Angelica sinensis, and Rehmannia glutinosa have been proven to ameliorate memory impairment [21–23]. *Cnidium monnieri* (L.) Cuss. shows effects of warming the kidney, activating the “yang” and blood, and is commonly used to treat kidney-Yang-deficient patients with the following symptoms: whitish tongue, fatigue, senescence, and impotence [24, 25]. Based on traditional Chinese medicine theory,* Cnidium monnieri* (L.) Cuss. is commonly used and ranked first *in “Shen Nong's Herbal Classic*.” Osthole (7-methoxy-8-isopentenoxy-coumarin), a major pharmacologically active constituent isolated from *Cnidium monnieri* (L.) Cuss., is a natural coumarin derivative. Research has implicated the role of osthole in combating erectile function [26] and has shown its properties of antiosteoporosis [27], antiproliferation [28], antiseizure [29], and antidiabetes [30]. Osthole also shows a neuroprotective effect on MPP^+^-induced cytotoxicity in PC12 cells [31]. Osthole also enhances the memory and ameliorates scopolamine- (Sco-) induced amnesia through its estrogen-like property in female rats [32, 33]. 

The enzymatic pocket of the HDACs was highly conserved. Therefore, most HDAC inhibitors act as paninhibitors. Knowledge on the contributions of individual HDAC family members is scant because of the lack of a specific HDAC inhibitor. In our study, osthole served as a source material that could be employed as a hydrophobic surface recognition cap for compound synthesis. A molecular docking analysis further suggested that the branched structure of osthole can provide isoforms with the selectivity of HDACs [34] to produce the active compound NBM-T-L-BMX-OS01 (BMX). BMX was identified as a potent inhibitor of HDAC8. This study shows that HDAC8 is highly expressed in the pancreas and brain. Because BMX enhances neurite outgrowth and CREB activation, the effects of BMX on neural plasticity such as learning and memory are examined in this study. 

## 2. Materials and Methods

### 2.1. Extraction and Isolation of Osthole

Seeds of *Cnidium monnieri* (L.) Cuss. (1 kg, dried weight) were purchased from a medicinal herb market in Taipei, Taiwan, in May 2008. The herb was extracted with acetone (3 × 10 L) at 25 ± 2°C for 2 weeks. After concentration of the combined extracts under reduced pressure, the residue (58.8 g) was suspended in H_2_O and then extracted with *n*-hexane : EtOAc = 70 : 30 (34.8 g). The extract (10 g) was chromatographed over a silica gel column and eluted with an *n*-hexane-EtOAc gradient system (10 : 0, 9 : 1, 8 : 2, and 7 : 3) to afford seven (F1–F4) fractions. The F3 (7.7 g) fraction was purified by HPLC (Phenomenex Luna C-18 silica gel column [10 × 250 mm], eluted with a mixture of MeOH and H_2_O [80 : 20, v/v]). Fractions containing osthole (**1**, 6.4 g) were collected at retention times of 7.4 min. ^1^H-NMR (400 MHz, CDCl_3_) 1.64 (3H, s, 5′), 1.81 (3H, s, 4′), 3.48 (2H, *d*, *J* = 7.3 Hz, 1′), 3.92 (3H, s, CH_3_O), 5.17 (1H, m, 2′), 6.20 (1H, *d*, *J* = 9.4 Hz, 4), 6.99 (1H, *d*, *J* = 8.7 Hz, 6), 7.43 (1H, *d*, *J* = 8.7 Hz, 5), 7.84 (1H, *d*, *J* = 9.4 Hz, 3), ^13^C-NMR (400 MHz, CDCl_3_) 16.09 (C4′), 20.8 (C1′), 24.0 (C5′), 54.7 (CH_3_O), 107.1 (C6), 111.2 (C4), 112.5 (C10), 116.7 (C8), 120.5 (C2′), 126.2 (C5), 131.4 (C3′), 144.3 (C3), 152.0 (C9), 159.9 (C7), 161.5 (C2). HREIMS *m*/*z* 244.30 (calcd. for C_15_H_16_O_3_ 244.29).

### 2.2. Preparation of 2-Hydroxy-3-prenyl-4-methoxy-trans-ethyl Cinnamate (**2**)

The solution of **1** (2.40 g, 10 mmol) in dry EtOH (20 mL) was added dropwise to the mixture of sodium ethoxide (1.36 g, 20 mmol) in dry EtOH (20 mL). The resulting solution was heated under nitrogen for 6 h and then diluted with dis-H_2_O (50 mL), acidified with 1 N HCl_(aq)_ to pH 4-5, extracted with EtOAc (50 mL × 3), and dried over Na_2_SO_4_. After removal of EtOAc under reduced pressure, the residue was purified with silica gel (EtOAc : *n*-hexane = 10 : 1) to yield **2**. ^1^H-NMR (500 MHz, CDCl_3_): *δ*7.92 (1H, *d*, *J* = 16.1 Hz), 7.33 (1H, *d*, *J* = 8.7 Hz), 6.49 (1H, *d*, *J* = 8.7 Hz), 6.44 (1H, *d*, *J* = 16.1 Hz), 6.10 (1H, s), 5.20 (1H, *t*, *J* = 7.0 Hz), 4.23 (2H, *q*, *J* = 7.2 Hz), 3.82 (3H, s), 3.42 (1H, *d*, *J* = 7.1 Hz), 1.82 (3H, s), 1.75 (3H, s), 1.31 (3H, *t*, *J* = 7.2 Hz).

### 2.3. Preparation of 2-(4-Methoxybenzoxy)-3-prenyl-4-methoxy-trans-ethyl Cinnamate (**3**)

Methoxy benzyl chloride (3.44 mmol) was added to the mixture of **2** (1.72 mmol) and K_2_CO_3_ (4.3 mmol) in acetone (20 mL). The resulting solution was heated under N_2_ overnight. After filtration to remove K_2_CO_3_, the filtrate was condensed under reduced pressure. The resulting residue was purified by gel (EtOAc : *n*-hexane = 15 : 1) to yield **3**. ^1^H-NMR (500 MHz, CDCl_3_): *δ*7.98 (1H, *d*, *J* = 16.1 Hz), 7.44 (1H, *d*, *J* = 8.7 Hz), 7.40 (2H, *d*, *J* = 8.4 Hz), 6.92 (2H, *d*, *J* = 8.4 Hz), 6.71 (1H, *d*, *J* = 8.7 Hz), 6.34 (1H, *d*, *J* = 16.1 Hz), 5.17 (1H, *t*, *J* = 6.4 Hz), 4.74 (2H, s), 4.25 (2H, *q*, *J* = 7.1 Hz), 3.86 (3H, s), 3.83 (3H, s), 3.39 (2H, *d*, *J* = 6.5 Hz), 1.73 (3H, s), 1.67 (3H, s), 1.33 (3H, *t*, *J* = 7.1 Hz).

### 2.4. Preparation of 2-(4-Methoxybenzoxy)-3-prenyl-4-methoxy-trans-Cinnamate (**4**)

The mixture of **3** (1.81 mmol) and 10% KOH/MeOH (20 mL) was refluxed overnight under N_2_ and then diluted with dis-H_2_O (100 mL), acidified with 2 N HCl to pH 5-6, and extracted with EtOAc (50 mL × 3). The combined EtOAc layer was dried over Na_2_SO_4_ and concentrated under a reduced pressure to yield **4**, termed NBM-T-L-BMX-OS01 (BMX). ^1^H-NMR (500 MHz, CDCl_3_): *δ*8.06 (1H, *d*, *J* = 16.1 Hz), 7.47 (1H, *d*, *J* = 8.7 Hz), 7.39 (2H, *d*, *J* = 8.5 Hz), 6.93 (2H, *d*, *J* = 8.5 Hz), 6.73 (1H, *d*, *J* = 8.7 Hz), 6.35 (1H, *d*, *J* = 16.0 Hz), 5.18 (1H, *t*, *J* = 6.5 Hz), 4.76 (2H, s), 3.88 (3H, s), 3.82 (3H, s), 3.39 (1H, *d*, *J* = 6.5 Hz), 1.73 (3H, s), 1.68 (3H, s).

### 2.5. HDACs Activity Assay

HDAC 1–11 enzyme activity was determined by Reaction Biology Corp. (Malvern, PA, USA). The substrate was fluorogenic peptide from p53 residue 379–382 (RHKKAc) and was measured with an excitation of 360 nm and an emission of 460 nm. BMX and trichostatin A (TSA, a pan-HDACi as a standard positive control) were tested in the platform. Both compounds were assayed in serial dilution concentrations from a starting concentration of 20 to 0.0009 *μ*M. Fluorescence intensity was measured using a fluorometric reader with an excitation of 360 nm and an emission of 460 nm. The EC_50_ value was present at the concentration of test compounds to inhibit 50% HDAC enzyme activity.

For the HDAC8 activity assay, the deacetylase activity of HDAC8 was measured using the commercially available Fluor-de-Lys-HDAC8 deacetylase substrate (Enzo, New York, NY, USA) according to the manufacturer instructions. In brief, the brain was dissected and was homogenized in PBS containing 0.32 M sucrose (pH 7.4) by using a Teflon glass homogenizer. The homogenate was centrifuged at 1000 ×g for 10 min to obtain the nuclei pellet. The resulting nuclei pellet was resuspended in a buffer containing 50 mM Tris-HCl, pH 8.0, 137 mM NaCl 2.7 mM KCl, and 1 mM MgCl_2_. To determine the HDAC activity, 10 *μ*g of nuclear extracts was incubated with a specific substrate (100 *μ*M) for 30 min at 37°C, followed by the addition of a developer solution for 30 min at 37°C. Fluorescence was measured using a Spectra Max Gemini XS fluorescent plate reader (Molecular Devices, Sunnyvale, CA, USA) with an excitation of 360 nm and an emission of 460 nm. Data were normalized to swimming control, footshock control, and nontrained-control animals.

### 2.6. Animals

Adult male Sprague-Dawley rats (250–350 g) were purchased from the National Laboratory Center in Taiwan. Rats were housed (2 per cage) in a temperature- (22–24°C) and humidity- (50%–60%) controlled room at the Animal Facility of Ilan University. Animals were housed in a room maintained on a 12 h/12 h light/dark cycle with food and water available ad libitum. Animals were allowed to acclimatize to the room for 1 week before any experimental procedure was conducted. All experimental procedures were approved by the Guide for the Care and Use of Laboratory Animals, by the National Institutes of Health (NIH Publications No. 8023, revised 1978), and were performed by people who had received the appropriate training by the National Laboratory Center in Taiwan. All experiments were also approved by the Ethical Committee of Animal Experimentation at Ilan University.

### 2.7. Drug Preparation and Treatment Schedule

SAHA was supplied by NatureWise Biotech and Medicals Corporation (Taipei, Taiwan). Scopolamine hydrochloride (Sco) was purchased from Sigma-Aldrich Co. (St. Louis, MO, USA). The BMX and SAHA stock (100 mg/mL) was dissolved in DMSO and diluted with saline to a final concentration of 0.5 mg/mL (in 0.5% DMSO/saline). Sco was dissolved in saline to a final concentration of 0.15 mg/mL based on previous studies [35, 36]. SD rats were randomly divided into 3 groups. For the naïve animal experiments, the BMX-injected or SAHA-injected groups were injected with BMX (5 mg/kg/d) or SAHA (5 mg/kg/d) intraperitoneally 7 d prior to behavioral experiments and during the experimentation. The control group received the 0.5% DMSO/saline injection. The injection volume was 10 *μ*L/g of body weight. The injections were performed once per day. For the amnesia experiment, the protocol was adopted from previous studies [35, 36] with modifications. The Sco-injected (SAHA + Sco) or (BMX + Sco)-injected groups were injected with Sco (1.5 mg/kg/d), SAHA (5 mg/kg/d) or BMX (5 mg/kg/d), intraperitoneally 7 d prior to behavioral experiments and during the experimentation. The control group received the 0.5% DMSO/saline injection. The injection volume was 10 *μ*L/g of body weight. The Sco injection was performed for 30 min before behavioral experiments, and the SAHA or BMX injection was conducted once daily at 5:00 PM 1 day prior.

### 2.8. RNA Extraction and Real-Time PCR

Total RNA from tissue was isolated by using the RNAspin Mini Kit (GE Healthcare, Waukesha, WI, USA). The cDNA was generated from total RNA with RevertAid Premium Reverse Transcriptase (Thermo Fisher Scientific, Waltham, MA, USA). Real-time PCR analysis was performed with the Rotor-Gene real-time PCR system (Qiagen, Hilden, Germany) by using the SYBR Green PCR Master Mix (Thermo Fisher Scientific) according to the instruction manual. The PCR parameters that were used are as follows: 95°C for 10 min for 1 cycle, 95°C for 15 s followed by 60°C for 1 min for 40 cycles.

The primer sequences for HDAC1 are as follows: forward: 5′-ATCGTCCTCACAAAGCCAAC-3′ and reverse: 5′-TGTCCGTCTGCTGCTTATTG-3′. The primer sequences for HDAC2 are as follows: forward: 5′-TGCTGTCCTCGAGCTACTGA-3′ and reverse: 5′-TCCCTCATGGGAAAGTTGAC-3′. The primer sequences for HDAC3 are as follows: forward: 5′-CTAGACCAGATCCGCCAGAC-3′ and reverse: 5′-TGGCCTGCTGTAGTTCTCCT-3′. The primer sequences for HDAC8 are as follows: forward: 5′-CGCTACCCCCGGTTTATATT-3′ and reverse: 5′-CTTCTTGGCTGACCTTCTGG-3′. These sequences were designed based on the Primer Design Program “Primer 3” software (http://frodo.wi.mit.edu/primer3/). The primer sequences for HPRT, forward: 5′-GCAGACTTTGC TTTCCTTGG-3′ and reverse: 5′-TCCACTTTCGCTGATGA CAC-3′.

### 2.9. Blood-Brain Barrier Penetration of BMX

Adult male SD rats (250–350 g) were anesthetized intraperitoneally with 1.0 g/mL of urethane and 0.1 g/mL of *α*-chloralose (1.0 mL/kg, i.p.). The assay was adopted from previous studies [37, 38] with modifications. The femoral veins were catheterized with PE-50 tubing for BMX (10 mg/kg) administration. Before dosing and 15 min later, the blood samples (0.3 mL) were collected, followed by brain tissue dissection. The brain stem, cerebellum, cerebral cortex, hippocampus, striatum, and the rest of the brain were dissected from the whole brain after 15 min of blood sampling. The plasma sample (50 *μ*L) was mixed with 150 *μ*L methanol and 5 *μ*L honokiol (10 *μ*g/mL, as internal control), followed by centrifugation at 16000 ×g for 10 min at 4°C. The supernatant (20 *μ*L) was analyzed using the HPLC-UV system. One gram of the brain sample was mixed with 5 mL 50% methanol and then homogenized at 15000 rpm. The homogenized solution was centrifuged at 4000 ×g for 10 min. The supernatant of the homogenized solution (50 *μ*L) was mixed with 5 *μ*L honokiol (10 *μ*g/mL, as internal standard) and 150 *μ*L methanol on a vortex for 10 s. The denatured protein precipitate was separated by centrifugation at 16000 ×g for 10 min at 4°C. The supernatant (20 *μ*L) of the sample solution was injected into the HPLC-UV system for analysis. Shimadzu liquid chromatography instrumentation (Nakakyo-ku, Kyoto, Japan) was used, which consisted of an LC-20AT pump, a DGU-20A5 on-line degasser, an SIL-20AC autosampler, and a SPD-M20A PDA detector. Separation was performed with an Agilent Zorbax extend-C18 (150 × 4.6 mm i.d., 5 *μ*m) column. The mobile phase was conducted with methanol: 10 mM KH_2_PO_4_ (pH 4.0) (78 : 22, v/v) at a flow rate of 1 mL/min. The detector wavelength was set at 300 nm. A stock solution (200 *μ*g/mL) of BMX was prepared in methanol and diluted with 50% methanol at concentrations of 0.1, 0.5, 1, 5, 10, 50, and 100 *μ*g/mL as a working solution. All 7-point calibration curves with concentrations of 0.01, 0.05, 0.1, 0.5, 1, 5, and 10 *μ*g/mL were prepared. Quality control samples for the determination of interday and intraday variations, accuracy, precision, and extraction recovery were prepared in the same manner as the calibration samples.

### 2.10. Primary Hippocampal Cultured Neurons

The primary hippocampal culture was prepared as described previously [39]. To culture embryonic hippocampal primary neurons, pregnant Sprague-Dawley rats were purchased from the National Laboratory Center in Taiwan. The hippocampal tissue from the embryos of the Sprague-Dawley rats (E19) was dissociated with 100 U/mL of papain (Sigma-Aldrich) and plated onto poly-L-lysine-coated coverslips at a density of 1 × 10^4^ cells/cm^2^ with a minimal essential medium containing 5% calf serum, 5% horse serum, and 50 ng/mL insulin-transferrin-selenite (Sigma-Aldrich). Three hours after plating, the medium was replaced with 2% of a B27-neurobasal medium (Invitrogen, Carlsbad, CA, USA) containing 0.5 mM glutamine and 12.5 *μ*M glutamate.

### 2.11. MTT Assay

Cell viability was evaluated by an MTT (3-(4,5-dimethylthiazol-2-yl)-2,5-diphenyl tetrazolium bromide, Sigma-Aldrich) assay 24 h after SAHA and BMX treatment at concentrations of 0, 1, 2.5, 5, and 10 *μ*M. The protocol was performed as described previously [40]. Cells were incubated with an MTT (0.5 mg/mL) reagent (Sigma-Aldrich) at 37°C for 1 h. The MTT solution was removed, and DMSO was added to the wells shaken at room temperature for 1 h. The amount of MTT formazan product was quantified by measuring its absorbance at 570 and 630 nm by using an ELISA plate reader (SpectraMax M2 Microplate Readers, Molecular Devices, Sunnyvale, CA, USA).

### 2.12. Western Blot

The hippocampus tissue and primary hippocampal cells were lysed and sonicated in an RIPA buffer (50 mM Tris-HCl (pH 7.4), 150 mM NaCl, 2 mM EDTA, 1% IGEPAL CA-630, 1 mM phenylmethylsulfonyl fluoride (PMSF), 20 *μ*g/mL pepstatin A, 20 *μ*g/mL leupeptin, 20 *μ*g/mL aprotinin, 50 mM NaF, and 1 mM Na_3_VO_4_). The protocol was performed as described previously [41]. The lysate was resolved by 8% SDS-PAGE. The proteins resolved by SDS-PAGE were transferred to the PVDF membrane (Millipore, Bedford, MA, USA) and western blotting was conducted with the following antibodies: rabbit anti-acetyl histone 4 lysine 12 and mouse anti-histone 4 (Cell signaling, Danvers, MA, USA). The secondary antibodies used include the HRP-conjugated goat-anti-rabbit IgG antibody and HRP-conjugated goat-anti-mouse IgG antibody (Millipore). The membrane was developed by reacting with a chemiluminescent HRP substrate (Millipore) and exposure to X-ray film. The protein bands were quantified using the NIH Image J Software.

### 2.13. Water-Maze Learning

The method was adopted from that of a previous study [41] with modifications. A plastic circular pool 2.0 m in diameter and 0.6 m in height was filled with water (25 ± 2°C) to a depth of 20 cm. A circular platform (8 cm in diameter) was placed at a specific location from the edge of the pool and submerged 2-3 cm below the water surface. Water was made cloudy by adding toxic-free dye. Distinctive visual cues were set on the wall. For spatial learning, animals were subjected to 3 trials per day, with one trial early in the morning, one trial at noon, and another in the late afternoon. The training procedure lasted 4 days, and a total of 12 trials were given. This procedure was adopted because spaced training is a better paradigm to facilitate memory consolidation. For these trials, the rats were positioned at different starting points spaced equally around the perimeter of the pool in random order. They had 120 s to find the hidden platform. If a rat could not find the platform, it was guided to the platform and was allowed to remain there for 20 s. The time each animal took to reach the platform was recorded as the escape latency. A probe trial of 120 s was given on day 5 to test their memory retention. The rats were placed in the pool with the platform removed, and the time they spent in each quadrant (Quadrants 1, 2, 3, and 4) was recorded. Quadrant 4 is the target quadrant. For the trained and swimming control experiments, rats in the trained group were subjected to the regular water maze learning procedure. At the end of the experiment, the rats were subjected to visible platform learning. For visible platform learning, a flag was mounted on the platform, and the platform was raised 2.5 cm above the surface of the water. In addition, the dye had not been added, so that the animals could see the location of the platform from the water. For each trial, the rats in the swimming control group swam for the same period as the trained group (use the mean latency value for each trial), with the difference of the visual cues and the platform having been removed.

### 2.14. Inhibitory Avoidance Learning Task

The apparatus consisted of a trough-shaped alley divided by a sliding door that separates an illuminated safety compartment and a dark compartment. A shock generator that produced current was connected to the floor of the dark compartment (UGO Basile, Comerio (VA), Italy). The method used was adopted from that of previous studies [42–44] with modifications. The behavioral task, including the training and testing procedures, was recorded between 8:00 AM and 6:00 PM. Before the experiment, the rats were habituated in a dim room for 1 h so that they could adjust to the environment. In the training phase, a rat was placed at the far end of the illuminated compartment facing away from the door. As the rat turned around, the door shut, and after 1 s, a 1 mA/s footshock was given twice. The rat was then removed from the alley and returned to its cage. At different times after training (1 d and 7 d later), the retention test was given. Rats were tested after 1 day and 7 days in the same manner as in the training, but without receiving a shock. Testing was terminated either when the rat entered the dark chamber or after 600 s without entry. Rats that did not enter the dark compartment and reached the ceiling score of 600 s were removed from the alley and assigned as rats with good memory [45]. The animals placed in the dark compartment who received footshock (1 mA/s for 1 s) directly were assigned to the footshock-only control group. 

### 2.15. Novel Object Recognition Learning

The method used was adopted from that of a previous study [46] with modifications. During familiarization, rats were allowed to explore 2 identical objects in an open field box (90 × 70 × 60 cm) for 5 min. The criteria used for exploration were a distance less than 1.5 cm between the rat and the object or direct contact with the object. During the retention test given 8 h later, the rats were returned to the same box, but one of the familiar objects was replaced with a novel object of approximately the same size. The time that each rat spent exploring the 2 objects during a 5 min period was recorded. The rats placed in the open field box without any objects for 5 min were assigned to the nontrained group.

### 2.16. Statistical Analysis

The biochemical data, part of water-maze data and passive one-way avoidance data, were analyzed with one-way analysis of variance (ANOVA), followed by post-hoc Newman-Keuls multiple-comparison test. The *q* values represent those calculated from Newman-Keuls analysis. Part of the water maze data and novel object recognition data was analyzed with two-way ANOVA, followed by post-hoc Newman-Keuls multiple-comparison test.

## 3. Results

### 3.1. NBM-T-L-BMX-OS01 (BMX) Is Semisynthesized from Osthole and Is a Potent HDAC8 Inhibitor

**Figure 1:**
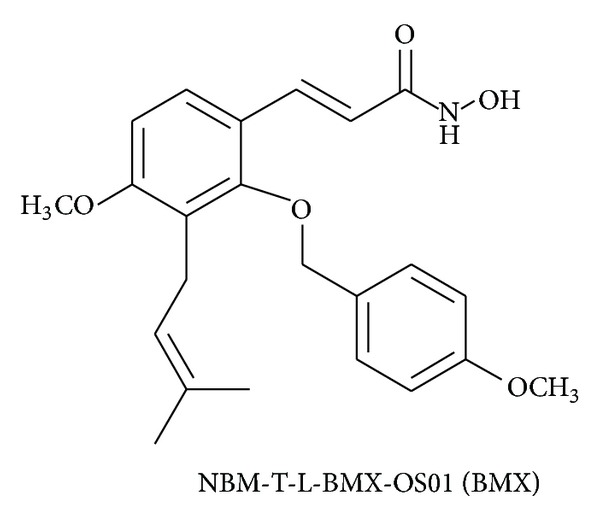
The NBM-T-L-BMX-OS01 (BMX) structure.

Osthole was isolated from *Cnidium monnieri* (L.) Cuss. and was roughly 2% of the dry weight of this herb. In this study, osthole served as a source material that, through 3 steps of semisynthesis, produced the active compound NBM-T-L-BMX-OS01 (BMX) (397.46 Da) (Figure 1). To examine whether BMX is an HDAC inhibitor (HDACi), the enzymatic activity of 11 HDACs was evaluated by BMX and TSA (a pan-HDACi as a positive control was used in the commercial assay kit). BMX inhibited HDAC8 and HDAC3 with EC_50_ 0.831 *μ*M and 27.3 *μ*M, respectively. TSA as a positive control is a pan-HDACi that significantly inhibited HDAC enzymes. BMX was 4963.6 times weaker in inhibiting HDAC3 enzyme activity than TSA (Table 1).

### 3.2. Tissue Distribution of HDAC8 mRNA

**Figure 2:**
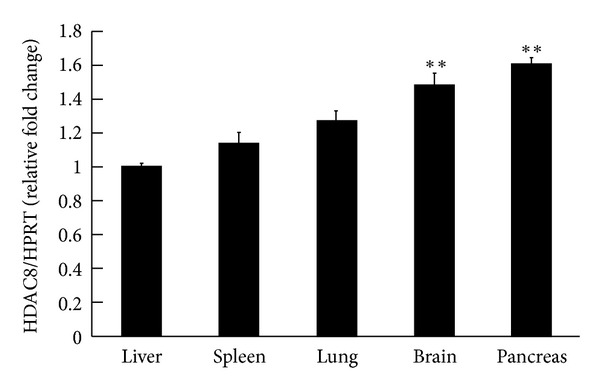
Level of HDAC8 mRNA in different tissues in naïve rats. Real-time PCR revealed that the pancreas and brain showed higher HDAC8 mRNA expression. The data are the mean ± SEM. ∗∗, in the comparison of the control group. **P < 0.01. One-way ANOVA followed by Newman-Keuls comparisons.

Naïve male SD rats (250–350 g) were euthanized, and the tissues were immediately dissected. Results from real-time PCR revealed that the pancreas and brain showed a higher HDAC8 mRNA expression (1.6-fold and 1.5-fold, *q* = 7.64 and 6.04, *P* < 0.01 compared to the liver group, resp. *N* = 4 in each group). Liver mRNA showed that the lowest expression was taken as 1. HPRT was used as an internal control (Figure 2).

### 3.3. Expression of HDAC1, -2, -3, and -8 mRNA in the Cortex, Hippocampus, and Amygdala

**Figure 3:**
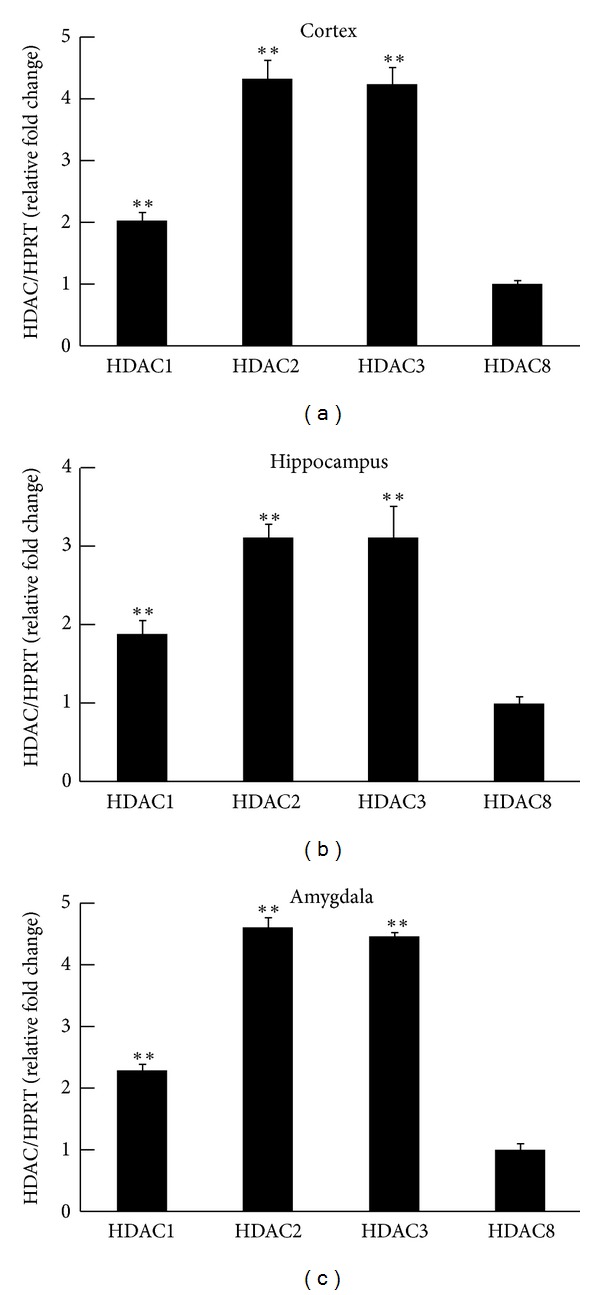
Level of HDAC8 mRNA in memory-related regions in naïve rats. Real-time PCR results showed that the expression of HDAC mRNA in the cortex is ranked HDAC2, HDAC3, HDAC1, and HDAC8, in that order, in the cortex (a), hippocampus (b), and amygdala (c). The data are the mean ± SEM. ∗∗, in the comparison of the control group. **P < 0.01. One-way ANOVA followed by Newman-Keuls comparisons.

The cortex, hippocampus, and amygdala are memory-related regions in the brain. Real-time PCR results showed that the expression of HDAC mRNA in the cortex is ranked HDAC2, HDAC3, HDAC1, and HDAC8, in that order (2.0-fold, 4.3-fold, and 4.2-fold, *q* = 11.92, 15.37, and 14.89, resp., all *P* < 0.01; compared to the HDAC8 group, resp. *N* = 8 in each group). HPRT was used as an internal control. HDAC8 mRNA showed that the lowest expression was taken as 1 (Figure 3(a)). Similar results were identified in the hippocampus and amygdala tissues. The expression of HDAC in the hippocampus is ranked HDAC2, HDAC3, HDAC1, and HDAC8, in that order (1.8-fold, 3.1-fold, and 3-fold, *q* = 4.25, 9.01, and 7.62, resp., all *P* < 0.01, compared to the HDAC8 group, resp. *N* = 8 in each group) (Figure 3(b)). The expression of HDAC in the amygdala is ranked HDAC2, HDAC3, HDAC1, and HDAC8, in that order (2.2-fold, 4.9-fold, and 4.6-fold, *q* = 4.81, 16.32, and 18.11, resp., all *P* < 0.01, compared to the HDAC8 group, resp. *N* = 8 in each group) (Figure 3(c)).

### 3.4. BMX Is Able to Penetrate the Blood-Brain Barrier (BBB)

**Figure 4:**
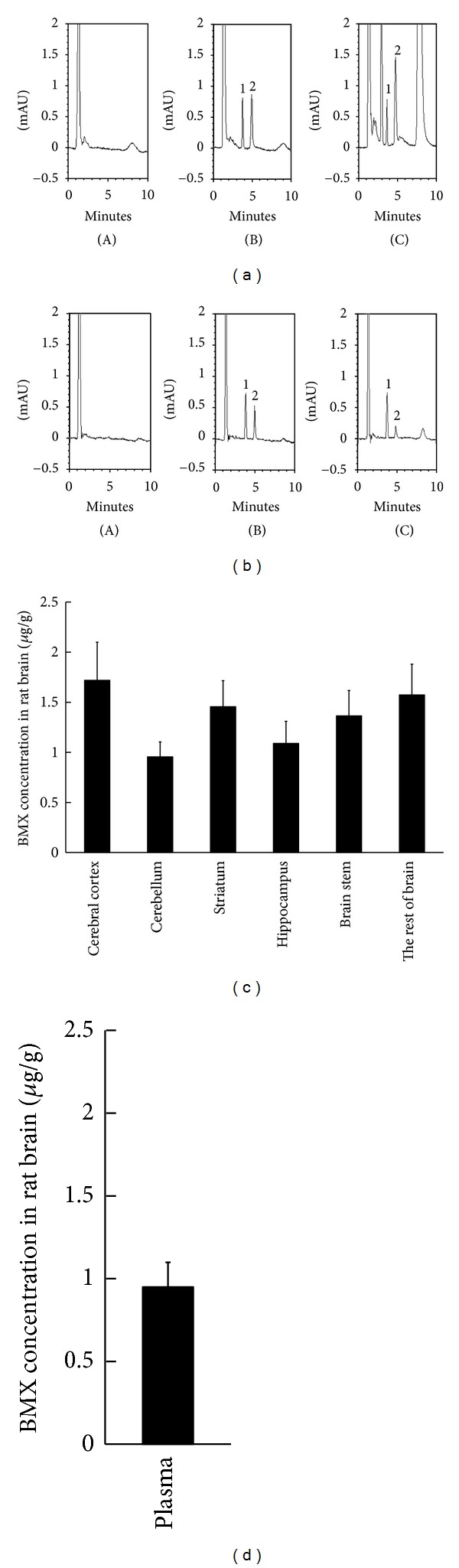
The BBB penetration assay of BMX. Chromatograms of plasma sample from (A) blank plasma; (B) blank plasma spiked with BMX (1 *μ*g/mL, internal standard) and honokiol (10 *μ*g/mL, internal standard); (C) plasma sample from rat plasma 15 min after honokiol (10 mg/kg, positive control, i.v.) and BMX (10 mg/kg, i.v.) administration. 1: honokiol; 2: BMX (a). Chromatograms of brain sample from (A) blank brain tissue; (B) blank brain spiked with BMX (1 *μ*g/mL, internal standard) and honokiol (10 *μ*g/mL, internal standard); (C) brain sample from the rat 15 min after honokiol (10 mg/kg, positive control, i.v.) and BMX (10 mg/kg, i.v.) administration. 1: honokiol; 2: BMX (b). Brain distribution profiles (c) and plasma concentration of BMX (d) 15 min after BMX (10 mg/kg, i.v.) was administered.

BMX (10 mg/kg) was intravenously (i.v.) injected into rats (each *N* = 6). The chromatograms of the plasma sample are as follows: Panel (A) was the blank plasma; Panel (B) was the blank plasma spiked with honokiol (10 *μ*g/mL, internal standard, Peak 1) and BMX (1 *μ*g/mL, Peak 2); and Panel (C) was the plasma sample from the rat plasma 15 min after honokiol and BMX (10 mg/kg, i.v.) administration (Figure 4(a)). After blood sampling, the brain tissue was dissected. Panel (A) was the blank brain tissue; Panel (B) was the blank brain tissue spiked with honokiol (10 *μ*g/mL, internal standard, Peak 1) and BMX (1 *μ*g/mL, internal standard, Peak 2); and Panel (C) was the brain tissue sample from the rats 15 min after honokiol and BMX (10 mg/kg, i.v.) administration (Figure 4(b)). BMX penetrated the BBB and was detected in brain areas including the cerebral cortex, cerebellum, striatum, hippocampus, brain stem, and the rest of the brain (Figure 4(c)). After brain dissection, the plasma sample was collected and assayed for BMX concentration (Figure 4(d)).

### 3.5. BMX Shows Less Neurotoxicity Than SAHA on Primary Hippocampal Neurons

**Figure 5:**
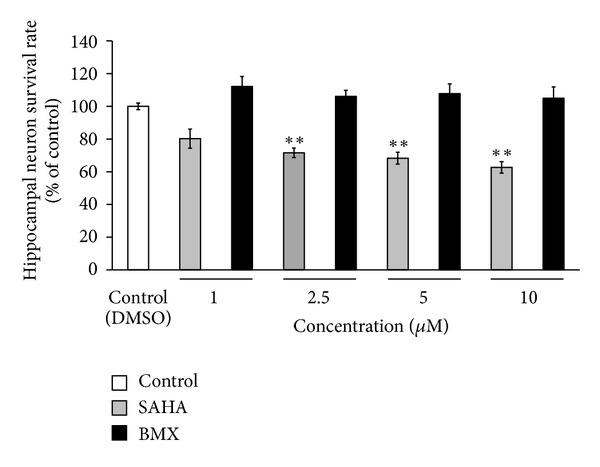
Effects of SAHA and BMX on primary hippocampal neuron viability by an MTT assay. SAHA and BMX (1, 2.5, 5, and 10 *μ*M) were applied to primary hippocampal neurons at DIV 5 for 24 h. SAHA reduced the survival rate of hippocampal neurons at concentrations of 2.5, 5, and 10 *μ*M. BMX showed little neurotoxic effect on the primary hippocampal neurons. The data are the mean ± SEM. ∗∗, in the comparison of the control group. **P < 0.01. One-way ANOVA followed by Newman-Keuls comparisons.

Using an MTT cell survival assay, SAHA reduced the survival rate of the hippocampal neurons at concentrations of 2.5, 5, and 10 *μ*M. BMX did not inhibit the survival rate of primary hippocampal neurons (Figure 5). BMX was shown to increase the neurite branch and neurite length in the primary hippocampal neurons. SAHA increased the neurite length in particular (see Supplementary Figure 1 available online at http://dx.doi.org/10.1155/2013/514908). Moreover, both SAHA and BMX increased CREB phosphorylation at Ser133 in hippocampal cultured neurons (Supplementary Figure 2(a)), as well as CRE-mediated promoter activity in HEK293T cells (Supplementary Figure 2(b)). These observations suggest that BMX might play a role in neural plasticity (e.g., learning and memory).

### 3.6. BMX and SAHA Enhance Spatial Learning

**Figure 6:**
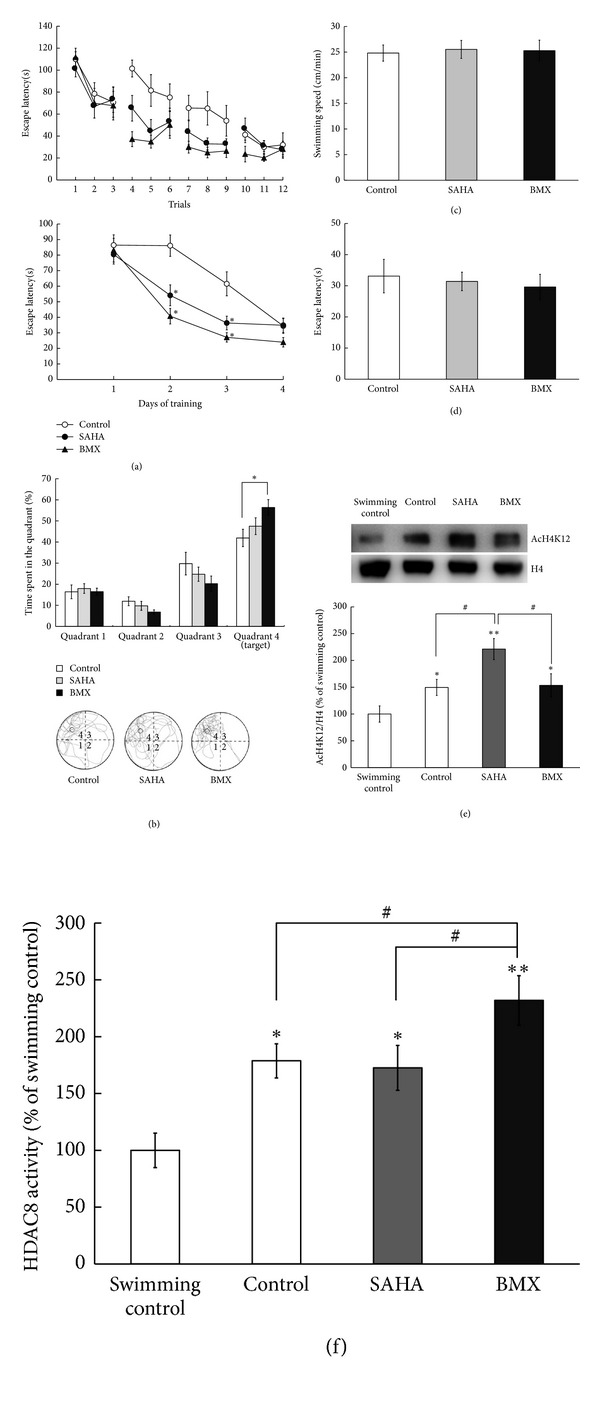
Effects of SAHA and BMX on water-maze memory acquisition and retrieval in rats. Control, SAHA-, and BMX-treated rats were subjected to water-maze training. The escape latency(s) of each water-maze learning trial ((a), upper) and the mean escape latency of each learning day ((a), lower) were recorded. On day 5, the hidden platform was removed, and the rats were allowed to swim for the probe trial test for 2 min. The time spent in each quadrant was recorded ((b), upper). The representative swimming pattern from the probe trial test is shown ((b), lower). We analyzed the swimming speed (c) and the escape latency of each water-maze learning trial in the visible platform (d) to rule out any motor, visual, or motivation side effect of BMX. After the experiment, the hippocampus was dissected for western blotting of histone 4 lysine 12 acetylation (AcH4K12). A representative gel pattern and quantitative analysis of AcH4K12 and the H4 level in the hippocampus (e). Quantitative analysis of HDAC8 activity in the hippocampus after the probe trial test (f). The data are the mean ± SEM. ∗, in the comparison of the control group; #, in the comparison between groups. ^∗,#^ *P* < 0.05; **P < 0.01. A two-way ANOVA followed by Newman-Keuls comparisons was applied for (a) and (b). A one-way ANOVA followed by Newman-Keuls comparisons was applied for (c), (d), (e), and (f).

Because of the ability of both BMX and SAHA [14] to penetrate the BBB, these drugs were administered intraperitoneally (5 mg/kg/d) to naïve rats (each *N* = 10). Rats receiving SAHA and BMX injections spent less time in finding the hidden platform (*F*_2,27_ = 4.94, *P* < 0.05). A marked decrease in escape latency was observed as early as Trial 4 in both BMX- and SAHA-treated groups (*q* = 6.59 and 3.71, *P* < 0.01, resp.) (Figure 6(a), upper). BMX and SAHA shortened escape latency, especially from day 2 (*q* = 4.81, *P* < 0.05, in a comparison of the control versus BMX-treated groups; *q* = 3.23, *P* < 0.05, in a comparison of the control versus SAHA-treated groups) (Figure 6(a), lower). Further probe trial tests for memory retrieval showed that rats receiving BMX spent more time in the quadrant where the platform had been placed (Quadrant 4 is the target quadrant) (*q* = 3.68, *P* < 0.05). SAHA-treated rats showed no difference compared with the control group (*P* > 0.05) (Figure 6(b), upper). The representative swimming patterns are shown in Figure 6(b) (lower). Swimming speed was also analyzed by a 2 min video recording in a pool without a platform to examine whether drugs have an effect on the motor activities of animals. During this recording, the speed is quantified as distance divided by time. Swimming speed was unaltered in all 3 groups (*F*_2,27_ = 0.04, *P* > 0.05) (Figure 6(c)) and in their visible platform learning performance (*F*_2,27_ = 1.1, *P* > 0.05) (Figure 6(d)). At the end of the experiment, the hippocampus was dissected for histone 4 lysine 12 acetylation (AcH4K12) and HDAC8 activity analysis. Because swimming itself might induce stress responses, and because stress has been reported to activate numerous signaling molecules, including HDAC [47], swimming control animals were taken as another control animal. Water-maze-trained-control animals showed higher H4K12 acetylation compared with the swimming control animals (*q* = 2.98, *P* < 0.05). SAHA-treated animals showed the highest H4K12 acetylation (*q* = 6.33, *P* < 0.01, compared with the swimming control animals), and BMX-treated animals showed similar H4K12 acetylation to trained-control animals (*q* = 2.88, *P* < 0.05, compared with the swimming control animals; *P* > 0.05, compared with the trained-control animals) (Figure 6(e)). Water-maze-trained-control animals showed higher HDAC8 activity compared with the swimming control animals (*q* = 4.07, *P* < 0.01). SAHA-treated animals did not increase HDAC8 activity (*q* = 0.32, *P* > 0.05, compared with the trained-control animals), and the BMX-treated animals showed increased HDAC8 activity (*q* = 3.01, *P* < 0.05, compared with the trained-control animals) (Figure 6(f)).

### 3.7. BMX, but Not SAHA, Enhances Passive One-Way Avoidance Tasks

**Figure 7:**
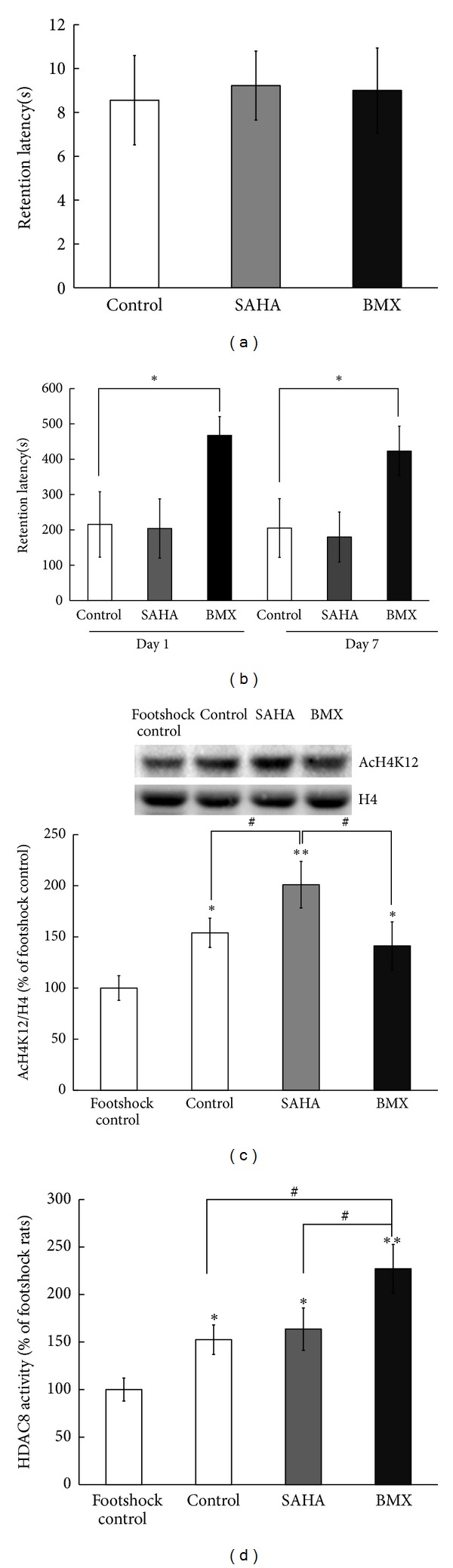
Effects of SAHA and BMX on passive one-way inhibitory avoidance learning in rats. Control, SAHA-, and BMX-injected rats were subjected to passive one-way inhibitory avoidance learning. Retention time in an illuminated compartment before footshock did not show any difference (a). Retention time in an illuminated compartment 1 day and 7 days after footshock was recorded (b). After the experiment, the amygdala was dissected for AcH4K12 western blotting. A representative gel pattern and quantitative analysis of AcH4K12 and the H4 level in the amygdala at 7 days after footshock (c). The HDAC8 activity in the amygdala 7 days after footshock was analyzed (d). The data are the mean ± SEM. ∗, in the comparison of the control group; #, in the comparison between groups. ^∗,#^ *P* < 0.05; **P < 0.01. One-way ANOVA followed by Newman-Keuls comparisons.

Naïve rats were randomly divided into control, SAHA, and BMX treatment groups (5 mg/kg/d, each *N* = 9). A one-trial footshock was delivered when rats entered a dark chamber from an illuminated chamber. Latency to enter the dark chamber 1 day and 7 days following footshock served as a measure of short-term and long-term memory retention. The cut-off latency was set at 600 s. Before receiving the footshock, all the rat groups spent approximately the same time entering the dark chamber from an illuminated chamber (*P* > 0.05, Figure 7(a)). Only BMX-treated rats showed significant retention time in the illuminated chamber 1 day after the footshock (*q* = 3.51, *P* < 0.05, in a comparison of the control versus BMX-treated groups on day 1) and 7 days after the footshock (*q* = 3.8, *P* < 0.05) (in a comparison of the control versus BMX group on day 7) (Figure 7(b)). No difference in retention performance between the control and SAHA groups was noticed (*P* > 0.05). At the end of the experiment, the amygdala was dissected for H4K12 acetylation and HDAC8 activity analysis. Rats that received only the footshock without training were taken as the footshock control animals. Trained-control animals showed higher H4K12 acetylation compared with the footshock-only control animals (*q* = 3.24, *P* < 0.05). SAHA-treated animals showed the highest H4K12 acetylation (*q* = 5.99, *P* < 0.01, compared with the footshock-only control animals), and the BMX-treated animals showed similar H4K12 acetylation to the trained-control group (*P* > 0.05, compared with the trained-control animals) (Figure 7(c)). Trained-control animals showed higher HDAC8 activity compared with the footshock-only control animals (*q* = 3.5, *P* < 0.05). SAHA-treated animals did not have increased HDAC8 activity (*P* > 0.05, compared with the trained-control animals), and the BMX-treated animals showed the highest HDAC8 activity (*q* = 3.62, *P* < 0.05, compared with the trained-control animals) (Figure 7(d)).

### 3.8. BMX Enhances Novel Object Recognition Tasks

**Figure 8:**
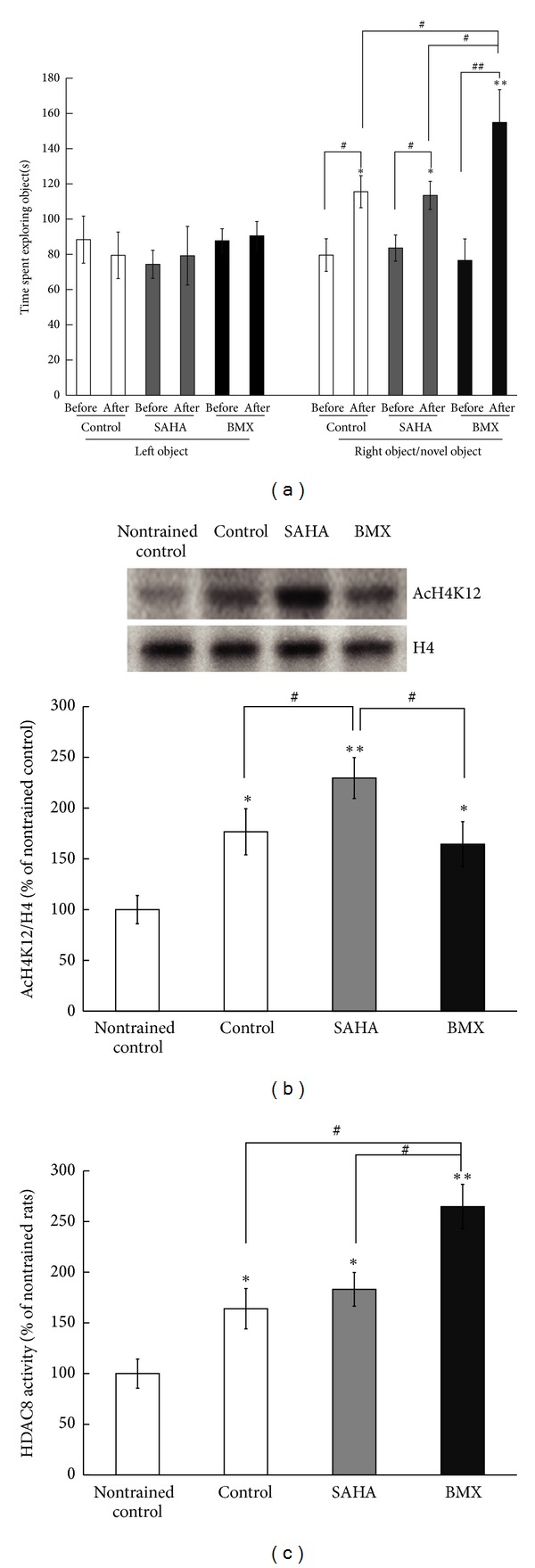
Effects of SAHA and BMX on the novel object recognition test in rats. Control, SAHA-, and BMX-injected rats were subjected to the novel object recognition test. The retention time(s) spent in exploring the left object, right object, and novel object before (pre-) and after (post-) the right object was replaced with the novel object was recorded (a). After the experiment, the hippocampus was dissected for AcH4K12 western blotting. A representative gel pattern and quantitative analysis of AcH4K12 and the H4 level from the rat hippocampus (b). The HDAC8 activity in the hippocampus was analyzed (d). The data are the mean ± SEM. ∗, in the comparison of the control group; #, in the comparison between groups. ^∗,#^ *P* < 0.05; ^∗∗,##^ *P* < 0.01. A two-way ANOVA followed by Newman-Keuls comparisons was applied for (a). A one-way ANOVA followed by Newman-Keuls comparisons was applied for (b) and (c).

In the novel object recognition task, the animals (each *N* = 9) did not show any preference for the left object (LO) or the right object (RO) during recognition training (pre-) (all *P* > 0.05) (Figure 8(a)). After Eight h (post-), a novel object (NO) replaced the RO. All rats showed a preference for the NO. Rats receiving BMX (5 mg/kg/d) showed a significant exploratory preference for the NO with respect to time spent exploring the object (Figure 8(a)) compared to the control and SAHA (5 mg/kg/d) treated rats (*q* = 4.47, *P* < 0.05; *q* = 4.51, *P* < 0.05). At the end of the experiment, the hippocampus was dissected for H4K12 acetylation and HDAC8 activity analysis. The animals placed in the open field box for 5 min without any objects were assigned to the nontrained group. The trained-control animals showed higher H4K12 acetylation compared with the nontrained animals (*q* = 3.61, *P* < 0.05). The SAHA-treated animals showed the highest H4K12 acetylation (*q* = 6.42, *P* < 0.01, compared with the nontrained animals), and BMX-treated animals showed similar H4K12 acetylation to the trained-control animals (*P* > 0.05, compared with the trained-control animals) (Figure 8(b)). The trained-control animals showed higher HDAC8 activity compared with the nontrained animals (*q* = 3.52, *P* < 0.05). The SAHA-treated animals did not have further increased HDAC8 activity (*P* > 0.05, compared with the trained-control animals), and the BMX-treated animals showed increased HDAC8 activity (*q* = 3.71, *P* < 0.05, compared with the trained-control animals) (Figure 8(c)).

### 3.9. BMX and SAHA Ameliorate Sco-Induced Memory Impairment in Spatial Learning

**Figure 9:**
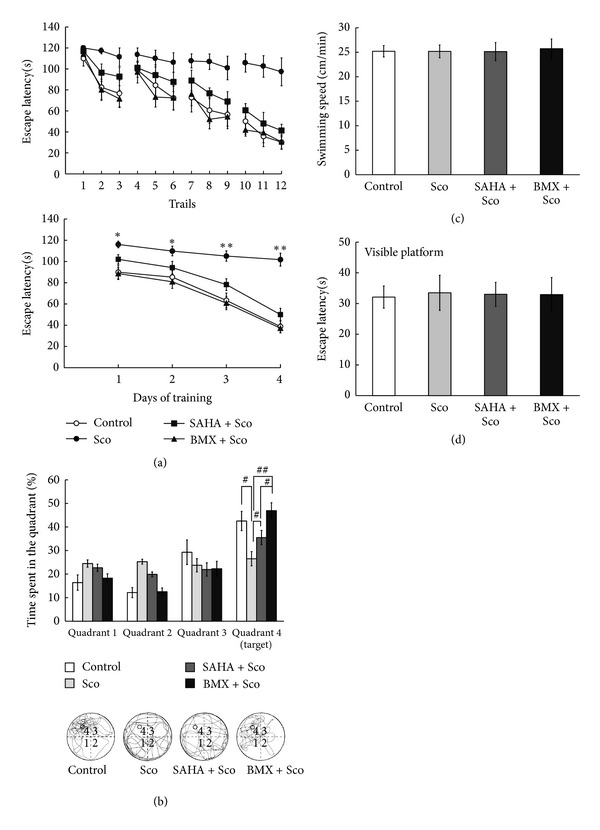
Effect of SAHA and BMX on Sco-induced memory impairment in water-maze memory acquisition and retrieval in rats. Control, scopolamine (Sco), (SAHA + Sco), and (BMX + Sco)-injected rats were subjected to water-maze training. The escape latency(s) of each water-maze learning trial ((a), upper) and the mean escape latency of each learning day ((a), lower) were recorded. On day 5, the hidden platform was removed, and the rats were allowed to swim for the probe trial test for 2 min. The time spent in each quadrant was recorded ((b), upper). The representative swimming pattern from the probe trial test is shown ((b), lower). We analyzed the swimming speed (c) and the escape latency of each water maze learning trial in the visible platform (d) to rule out any motor, visual, or motivation side effect of drugs. The data are the mean ± SEM. ∗, in the comparison of the control group; #, in the comparison between groups. ^∗,#^ *P* < 0.05; ^∗∗,##^ *P* < 0.01. A two-way ANOVA followed by Newman-Keuls comparisons was applied for (a) and (b). A one-way ANOVA followed by Newman-Keuls comparisons was applied for (c) and (d).

The treatment of Sco, a muscarinic receptor antagonist in animals and humans, causes impairments in learning and memory [48]. The Sco-induced amnesia animal model is frequently used in research on aging and dementia [23, 35, 49, 50]. Sco (1.5 mg/kg/d) and BMX (5 mg/kg/d) were injected for 7 days prior to the behavioral test and persisted to be injected during the experimentation (each *N* = 10). Rats receiving Sco injection showed longer escape latency in finding the hidden platform (*F*_3,36_ = 18.75, *P* < 0.01). A marked improvement in escape latency was observed as early as Trial 2 in (BMX + Sco)-treated groups (*q* = 4.02, *P* < 0.05) (Figure 9(a), upper). BMX attenuates Sco-induced longer escape latency, especially from day 1 (*q* = 3.36, *P* < 0.05, in a comparison of the Sco versus (BMX + Sco)-treated groups) (Figure 9(a), lower). An improvement in escape latency was observed from Trial 8 in (SAHA + Sco)-treated groups (*q* = 3.46, *P* < 0.05) (Figure 9(a), upper). SAHA attenuates Sco-induced longer escape latency, especially from day 3 (*q* = 3.89, *P* < 0.05, in a comparison of the Sco versus (SAHA + Sco)-treated groups) (Figure 9(a), lower). Further probe trial tests for memory retrieval showed that rats receiving Sco spent less time in the quadrant where the platform had been placed (Quadrant 4 is the target quadrant) (*q* = 5.79, *P* < 0.01, when compared with control group). (BMX + Sco)-treated and (SAHA + Sco)-treated rats spent more time in the target quadrant (*q* = 7.39, *P* < 0.01; *q* = 3.26, *P* < 0.05, when compared with Sco-treated group) (Figure 9(b), upper). The representative swimming patterns are shown in Figure 9(b) (lower). Swimming speed was unaltered in all 4 groups (*P* > 0.05) (Figure 9(c)) and in their visible platform learning performance (*P* > 0.05) (Figure 9(d)).

### 3.10. BMX Ameliorates Sco-Induced Memory Impairment in Passive One-Way Avoidance Tasks

**Figure 10:**
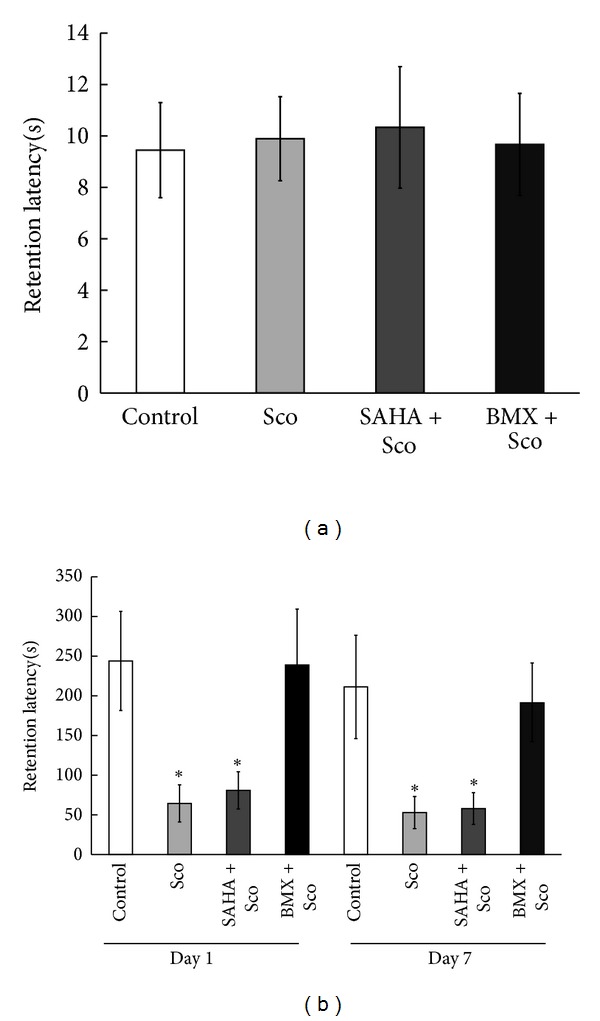
Effect of SAHA and BMX on Sco-induced memory impairment in passive one-way inhibitory avoidance learning in rats. Control, scopolamine (Sco), (SAHA + Sco), and (BMX + Sco)-injected rats were subjected to passive one-way inhibitory avoidance learning. Retention time in an illuminated compartment before footshock did not show any difference (a). Retention time in an illuminated compartment 1 day and 7 days after footshock was recorded (b). The data are the mean ± SEM. ∗, in the comparison of the control group. *P < 0.05. One-way ANOVA followed by Newman-Keuls comparisons.

Naïve rats were randomly divided into control, Sco, (SAHA + Sco), and (BMX + Sco) treatment groups (each *N* = 8). Sco (1.5 mg/kg/d), SAHA (5 mg/kg/d), or BMX (5 mg/kg/d) was injected for 7 days prior to the behavioral test and persisted to be injected during the experimentation. Before receiving the footshock, all the rat groups spent approximately the same time entering the dark chamber from an illuminated chamber (*P* > 0.05, Figure 10(a)). Sco-treated rats showed less retention time in the illuminated chamber 1 day after the footshock (*q* = 4.12, *P* < 0.05, in a comparison of the control versus Sco-treated groups on day 1) and 7 days after the footshock (*q* = 4.3, *P* < 0.05) (in a comparison of the control versus Sco-treated group on day 7). (SAHA + Sco)-treated rats showed less retention time in the illuminated chamber 1 day after the footshock (*q* = 3.12, *P* < 0.05, in a comparison of the control versus (SAHA + Sco)-treated groups on Day 1) and 7 days after the footshock (*q* = 3.9, *P* < 0.05) (in a comparison of the control versus (SAHA + Sco)-treated group on Day 7). No difference in retention performance between the control and (BMX + Sco) groups was noticed (*P* > 0.05). (BMX + Sco)-treated rats spent more time in the illuminated chamber 1 day after the footshock (*q* = 4.53, *P* < 0.05, when compared with Sco-treated group) and 7 days after the footshock (*q* = 3.51, *P* < 0.05, when compared with Sco-treated group) (Figure 10(b)).

### 3.11. BMX Ameliorates Sco-Induced Memory Impairment in Novel Object Recognition Tasks

**Figure 11:**
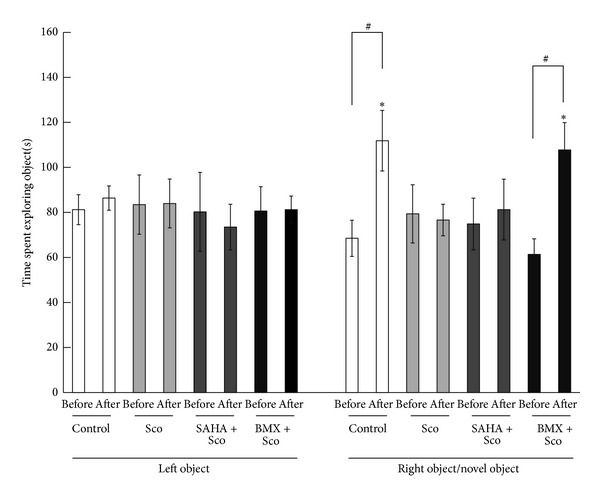
Effect of SAHA and BMX on Sco-induced memory impairment in the novel object recognition test in rats. Control, scopolamine (Sco), (SAHA + Sco), and (BMX + Sco)-injected rats were subjected to the novel object recognition test. The retention time(s) spent in exploring the left object, right object, and novel object before (pre-) and after (post-) the right object was replaced with the novel object was recorded. The data are the mean ± SEM. ∗, in the comparison of the control group; #, in the comparison between groups. ^∗,#^ *P* < 0.05. A two-way ANOVA followed by Newman-Keuls comparisons was applied.

In the novel object recognition task, Sco (1.5 mg/kg/d) and BMX (5 mg/kg/d) were injected for 7 days prior to the behavioral test and persisted to be injected during the experimentation. The animals (each *N* = 8) did not show any preference for the left object (LO) or the right object (RO) during recognition training (pre-) (all *P* > 0.05). After Eight h (post-), a novel object (NO) replaced the RO. Control and (BMX + Sco)-treated rats showed a preference for the NO (*q* = 3.79, *P* < 0.05; *q* = 3.92, *P* < 0.05). Rats receiving Sco or (SAHA + Sco) injection showed no exploratory preference for the NO with respect to time spent in exploring the RO (*P* > 0.05) (Figure 11).

## 4. Discussion

This study reports on a potent HDAC8 inhibitor BMX with little neurotoxicity, but with great potential for memory improvement. Our study shows that HDAC8 is highly expressed in the pancreas and the brain. The expression level of HDAC in memory-related regions, including the cortex, hippocampus, and amygdala, is ranked HDAC2, HDAC3, HDAC1, and HDAC8, in that order. Although the expression of HDAC8 is the lowest, the loss of HDAC8 causes embryonic lethality [51]. The HDAC8-selective inhibitor suppressed neuroblastoma cell proliferation [17, 52]. Knockdown of HDAC8 initiates neuronal differentiation such as an outgrowth of the neurite-like structure in human neuroblastoma cell lines [52]. BMX, a potent HDAC8 inhibitor, shows less cytotoxicity and induces significant neurite outgrowth including neurite branch and length in primary hippocampal neuron culture (Supplementary Figure 1). In Supplementary Figure 2, BMX is shown to increase CREB phosphorylation at Ser133 and also enhance CRE-mediated promoter activity. CREB and CRE-mediated gene expression levels are necessary for memory formation in many studies [53]. Neural plasticity, including neurite outgrowth and CREB activation, is necessary for long-term memory formation. Taken together, this suggests a role of BMX in memory formation and neural plasticity.

This is the first study to search for the role of the osthole-derived compound BMX in memory formation. To examine declarative and nondeclarative memory, a water maze, a passive one-way avoidance task, and a novel object recognition task were performed. The Morris water maze is a paradigm for spatial memory formation, which is an early deficit of Alzheimer's disease. In the water-maze task, BMX-treated rats even showed significant memory retrieval performance compared to SAHA-treated rats in the probe trial test. Neither BMX nor SAHA affects their visual discrimination ability, motor coordination, and motivational state. The water maze is a task that relies on the hippocampal function. The dorsal hippocampus is critical for several types of memory, including spatial and working memory [54], and it has been recently implicated in object recognition memory [55].

Numerous aversive conditioning procedures have been used to study memory regarding emotional experiences in rats. Passive-avoidance response is extensively used to screen drugs affecting learning and memory. The amygdala and other connected regions are critical for passive-avoidance learning. The training procedure comprises a single trial and is based on the innate preference of rodents for a dark chamber and the avoidance of punishment. The application of strong electric shock (1 mA or higher) is an appropriate method to examine memory retention *in vivo* [42–44]. In our experiment, an electric footshock (1 mA) was delivered. We found that BMX-treated, but not SAHA-treated, rats stayed longer in the illuminated chamber, and this effect lasted at least 7 days.

The novel object recognition test has become popular for testing recognition memory in rodents and for testing the effects of amnesic drugs. It is based on the premise that rodents explore a novel object more than a familiar one, but only if they remember the familiar one. This tendency is shared by human declarative memory [56]. The results of the novel object recognition task revealed that only BMX-treated rats spend more exploratory time on the novel object 8 h later. Transcription of immediate-early genes (e.g., *Arc*) triggers the labile memory to lasting memory in the modification of neuronal networks [57]. For example, an *Arc* mRNA increase occurs at least for 8 h in the hippocampal formation following a spatial behavioral experience [58]. Whether BMX plays a unique role in transforming fragile memory into long-term memory must be further studied. Other HDAC inhibitors such as sodium butyrate also transform a labile learning event into a remembered long-term event in the novel object recognition test [59].

In addition, BMX not only facilitates learning and memory in naïve animals, but also ameliorates Sco-induced memory impairment. The Sco-induced amnesia rats were adopted to evaluate the antiamnesic efficiency [49]. The animals injected with Sco featured impaired memory performance in the Morris water-maze test, novel object recognition test, and step-through passive avoidance test [36, 60, 61]. In addition, the administration of Sco has been proposed as a pharmacological model for Alzheimer's disease. The administration of Sco results in the increase of A*β* generation and *γ*-secretase activity in the cortex and hippocampus [60, 62]. In addition, Sco triggers oxidative stress in the brain of rats [63, 64]. This strengthens the value of BMX in treating memory-related diseases.

Class I HDAC inhibitors such as sodium valproate, sodium butyrate, and SAHA reversed contextual fear-conditioning memory in APPswe/PS1dE9 Alzheimer's disease mice at a concentration of 100 mg/kg for 2 weeks [15], which might have caused unexpected side effects at such a high concentration. For example, TSA, with a basal toxicity and prolonged treatment at high doses, often induces neuronal death, thus compromising their neuroprotective effect [65]. Sodium butyrate inhibits HDAC1, -2, -3, -6, -8, and -10 causing cell apoptosis [66]. Valproic acid, which inhibits HDACs 1–4 and 5–9, stops the entry of the G1 phase and also leads to cell apoptosis [67]. SAHA might damage neuronal cells through inhibition of HDAC1 or HDAC2 enzyme activity which cannot be ruled out [6, 17–20]. Our findings reveal that BMX did not cause a neurotoxic effect, whereas SAHA, at a concentration of 2.5 *μ*M, reduces cell survival in primary hippocampal neurons (Figure 5). Because of the possibility of cell damage from pan-HDAC inhibitors, BMX can be valued for its safety and isoform-selective property.

In an HDAC1–11 activity assay, although BMX inhibits HDAC3 and −8, we proposed that BMX should only inhibit HDAC8 *in vivo* at the concentration (5 mg/kg) we administered. As shown in Figures 4(c) and 4(d), BMX was detected in the brain at an average concentration of approximately 1.2 *μ*g/g (1.2 ppm), and the plasma concentration was approximately 1 *μ*g/mL (3 *μ*M) 15 min after intravenous (10 mg/kg) administration. In other words, for the BMX injected at 5 mg/kg as in our current behavior study, we proposed that the plasma BMX concentration should be less than 1.5 *μ*M in 15 min, at which the concentration that blocks HDAC8 activity (EC_50_ = 0.831 *μ*M) may exert only a slight effect on HDAC3 activity (EC_50_ = 27.3 *μ*M). This was partially confirmed by the results that showed that BMX did not affect Histone 4 acetylation by western blotting. If BMX inhibits HDAC3, H4K12 acetylation should have raised. This is also congruent with a previous report that the HDAC8-selective inhibitor did not alter histone acetylation [17, 52] and *α*-tubulin acetylation [68]. The substrate of HDAC8 *in vivo* remains unknown. Conversely, SAHA was reported to restore H4K12 acetylation loss in aged mice [13]. SAHA inhibits HDAC8 activity only at a relatively high concentration [16].

The molecular mechanism underlying the effect of BMX on enhancing memory formation must still be investigated. Two HDAC8 inhibitors, PCI34051 and Chrysin, which were identified recently, were reported to inhibit tumor growth [68, 69]. BMX shows similar EC50 as PCI34051 on HDAC8 activity inhibition in our unpublished data. To date, few substrates for HDAC8 *in vivo* have been identified. HDAC8 forms a complex with PP1 and CREB and functions to inactivate CREB-mediated gene transcription [70]. This suggests that BMX may modulate CREB-associated proteins or genes to contribute to memory formation and neural plasticity. Further studies such as microarray or proteomic studies should be conducted to identify the BMX substrate and its downstream signaling molecules involved in learning and memory.

## Supplementary Material

Supplementary Results: BMX was shown to increase the neurite branch and neurite length in the primary hippocampal neurons. SAHA increased the neurite length in particular (Supplemental Figure 1). Moreover, both SAHA and BMX increased CREB phosphorylation at Ser133 in hippocampal cultured neurons (Supplemental Figure 2(a)), as well as CRE-mediated promoter activity in HEK293T cells (Supplemental Figure 2(b)).

## Figures and Tables

**Table 1:** The inhibition assay of HDACs activities by NBM-T-L-BMX-OS01 (BMX) and trichostatin A (TSA).

HDACs (EC_50_, M)	HDACis	
	NBM-T-L-BMX-OS01 (BMX)	Trichostatin A (TSA)
HDAC1	—	3.37 × 10^−9^
HDAC2	—	7.14 × 10^−9^
HDAC3	2.73 × 10^−5^	5.55 × 10^−9^
HDAC4	—	9.51 × 10^−8^
HDAC5	—	6.99 × 10^−9^
HDAC6	—	9.96 × 10^−10^
HDAC7	—	2.46 × 10^−8^
HDAC8	8.31 × 10^−7^	1.31 × 10^−7^
HDAC9	—	1.39 × 10^−8^
HDAC10	—	1.14 × 10^−8^
HDAC11	—	6.15 × 10^−9^
