# Supplementary material for: NBM-T-L-BMX-OS01, Semisynthesized from Osthole, Is a Novel Inhibitor of Histone Deacetylase and Enhances Learning and Memory in Rats
Source: Evid Based Complement Alternat Med. 2013 Mar 28;2013:514908. doi: 10.1155/2013/514908 (PMC3625590; doi:10.1155/2013/514908)
Supplement: Supplementary file 1 — Supplementary Results: BMX was shown to increase the neurite branch and neurite length in the primary hippocampal neurons. SAHA increased the neurite length in particular (Supplemental Figure 1). Moreover, both SAHA and BMX increased CREB phosphorylation at Ser133 in hippocampal cultured neurons (Supplemental Figure 2(a)), as well as CRE-mediated promoter activity in HEK293T cells (Supplemental Figure 2(b)). [file 514908.f1.pdf]

Supplemental FIGURE 1

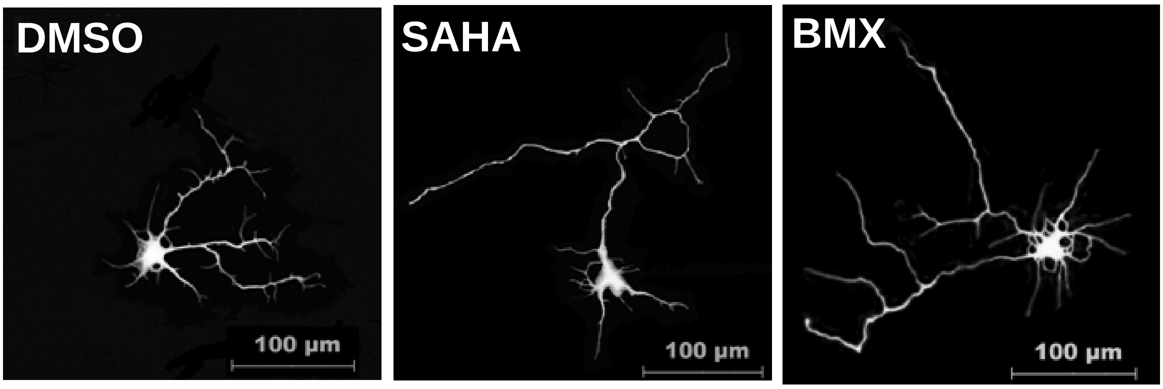

Supplemental FIGURE 2

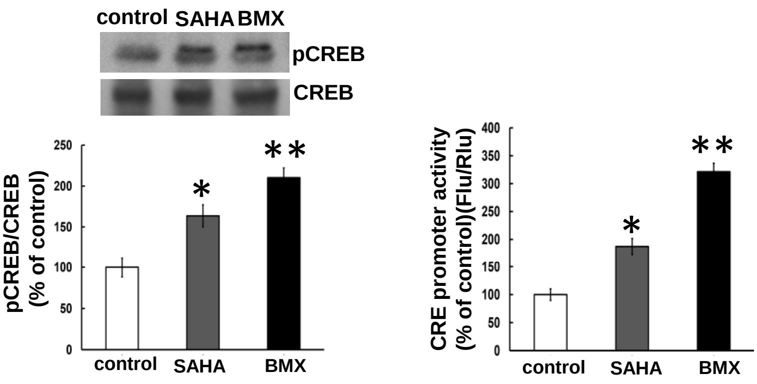

(a) (b)

Supplemental Materials and Methods

2.1. HEK293T cells. HEK293T cells were maintained in Dulbecco's modified Eagle's medium (Invitrogen) supplemented with 10% fetal calf serum (Invitrogen). Cultures were maintained in a humidified 5% CO2-95% air atmosphere at 37°C.

2.2. Immunocytochemistry. Cultured hippocampal neurons were fixed with 4% paraformaldehyde for 15 min and permeablized with 0.1% Triton X-100 for 20 min at room temperature (RT). The primary Mouse anti-β III tubulin and anti-actin (Millipore) Abs were added for 1 h at RT. After wash, Alexa Fluor 488 goat anti-rabbit Ab (Molecular Probes, Europe BV, Leiden, Netherlands) were incubated with cells for 1 h at RT. Coverslips were mounted, and images were obtained by using a Axio Observer D1 microscope (Zeiss, Jena, Germany). Neuronal process longer than 10 μM is defined as neurites.

2.3. Western blot. The hippocampus tissue and primary hippocampal cells were lysed and sonicated in RIPA buffer (50 mM Tris-HCl (pH 7.4), 150 mM NaCl, 2 mM EDTA, 1% IGEPAL CA-630, 1 mM phenymethylsulfonyl fluoride (PMSF), 20 μg/ml pepstatin A, 20 μg/ml leupeptin, 20 μg/ml aprotinin, 50 mM NaF and 1 mM Na_3VO_4). The lysate was resolved by 8% SDS-PAGE. The proteins resolved by SDS-PAGE were transferred to the PVDF membrane (Millipore) and western blot was conducted by using the following antibodies: rabbit anti-acetyl histone 4 Lysine12 and mouse anti-histone 4 (Cell signaling, Danvers, MA, USA). The secondary antibodies used include HRP-conjugated goat anti-rabbit IgG antibody and HRP-conjugated goat anti-mouse IgG antibody (Millipore). Membrane was developed by reacting with chemiluminescent HRP substrate (Millipore) and exposed to X-ray film (Kodak). The protein bands were quantified using the NIH Image J Software.

2.4. Plasmid construction. p3×CRE-Luc, which was constructed by cloning three CREs in front of the SV40 late promoter and the following firefly luciferase gene of pGL2-Promoter plasmid (Promega, Madison, HI, USA) according to Chen et al. (1)

2.5. Transfection. For HEK293T cells, Lipofectamine 2000 reagent (Invitrogen) was used for the transfection of plasmids as manual suggested.

2.6. Promoter-luciferase assay. HEK 293T cells seeded at a density of 1×10^4 cells per well in 12-well clusters were co-transfected with 0.5 μg promoter-firefly luciferase plasmid, 50 ng Renilla luciferase-encoding internal control plasmid phRG-TK, 1 μg

expression plasmid. After 24 h, medium was replaced with DMEM containing 10% FBS. Cells were incubated overnight and then subjected to luciferase activity assay by using the Dual-Glo luciferase assay system (Promega) and the Lumat LB9507 (Berthold Technologies, Bad Wildbad, Germany). Briefly, cells were trypsinized and washed with phosphate-buffered saline (PBS) twice. Cells were suspended in 50 lL PBS and added to 96-well microplate for luciferase assay. The activity of firefly luciferase was measured as firefly luciferase luminescence after reaction with luciferase reagent for 15 min. Next, the activity of Renilla luciferase is measured as Renilla luciferase luminescence after reaction with Stop & Glo reagent for 15 min. The ratio of firefly luciferase activity to Renilla luciferase activity was calculated and normalized to vector control.

Reference

1. Chen W, Yu YL, Lee SF, et al. "CREB is one component of the binding complex of the Ces-2/E2A-HLF binding element and is an integral part of the interleukin-3 survival signal," *Mol Cell Biol, vol.* 21, no. 14, pp. 4636-4646.
